# Genomic conservation of cattle microsatellite loci in wild gaur (*Bos gaurus*) and current genetic status of this species in Vietnam

**DOI:** 10.1186/1471-2156-8-77

**Published:** 2007-11-06

**Authors:** Trung Thanh Nguyen, Sem Genini, Linh Chi Bui, Peter Voegeli, Gerald Stranzinger, Jean-Paul Renard, Jean-Charles Maillard, Bui Xuan Nguyen

**Affiliations:** 1 Vietnamese Academy of Sciences and Technology, Hanoi, Vietnam; 2 Parco Tecnologico Padano (PTP), CERSA, Via Einstein, 26900 Lodi, Italy; 3 Institute of Animal Sciences, Breeding Biology, Swiss Federal Institute of Technology, 8092 Zurich, Switzerland; 4 UMR Biologie du Développement et de la Reproduction. INRA, 78350 Jouy en Josas, France; 5 Centre de Coopération Internationale en Recherche Agronomique pour le Développement (CIRAD), Montpellier, France

## Abstract

**Background:**

The wild gaur (*Bos gaurus*) is an endangered wild cattle species. In Vietnam, the total number of wild gaurs is estimated at a maximum of 500 individuals. Inbreeding and genetic drift are current relevant threats to this small population size. Therefore, information about the genetic status of the Vietnamese wild gaur population is essential to develop strategies for conservation and effective long-term management for this species. In the present study, we performed cross-species amplification of 130 bovine microsatellite markers, in order to evaluate the applicability and conservation of cattle microsatellite loci in the wild gaur genome. The genetic diversity of Vietnamese wild gaur was also investigated, based on data collected from the 117 successfully amplified loci.

**Results:**

One hundred-thirty cattle microsatellite markers were tested on a panel of 11 animals. Efficient amplifications were observed for 117 markers (90%) with a total of 264 alleles, and of these, 68 (58.1%) gave polymorphic band patterns. The number of alleles per locus among the polymorphic markers ranged from two to six. Thirteen loci (*BM1314*, *BM2304*, *BM6017*, *BMC2228*, *BMS332*, *BMS911*, *CSSM023*, *ETH123*, *HAUT14*, *HEL11*, *HEL5*, *ILSTS005 *and *INRA189*) distributed on nine different cattle chromosomes failed to amplify wild gaur genomic DNA. Three cattle Y-chromosome specific microsatellite markers (*INRA124*, *INRA126 *and *BM861*) were also highly specific in wild gaur, only displaying an amplification product in the males. Genotype data collected from the 117 successfully amplified microsatellites were used to assess the genetic diversity of this species in Vietnam. Polymorphic Information Content (PIC) values varied between 0.083 and 0.767 with a mean of 0.252 while observed heterozygosities (*H*_*o*_) ranged from 0.091 to 0.909 (mean of 0.269). Nei's unbiased mean heterozygosity and the mean allele number across loci were 0.298 and 2.2, respectively.

**Conclusion:**

Extensive conservation of cattle microsatellite loci in the wild gaur genome, as shown by our results, indicated a high applicability of bovine microsatellites for genetic characterization and population genetic studies of this species. Moreover, the low genetic diversity observed in Vietnamese wild gaur further underlines the necessity of specific strategies and appropriate management plans to preserve this endangered species from extinction.

## Background

The wild gaur, also known as the Indian bison or seladang, is a member of the subfamily Bovinae and is currently classified among endangered species and listed as vulnerable by International Union for Conservation of Nature and Natural Resources [1]. According to the Asian Wild Cattle Conservation Assessment and Management Plan (CAMP – [2]), three wild subspecies are generally recognized, including *Bos gaurus laosiensis *(Myanmar to China), *Bos gaurus hubbacki *(Thailand and Malaysia) and *Bos gaurus gaurus *(India and Nepal). Recently, the species name *Bos gaurus *was suggested for wild gaur instead of *Bibos gauris *or *Bos frontalis *by the International Commission on Zoological Nomenclature [3]; this name is currently used.

The gaur is one of the most impressive and largest of the wild cattle. A typical adult wild gaur bull may measure up to two meters at the shoulders and 900 kg in weight [4]. Gaurs are gregarious animals that live in hilly terrains below an altitude of 1,800 meters in herds ranging from 6 to 40 individuals. The distribution of wild gaur includes areas of southern and south-eastern Asia, from India to peninsular Malaysia, occurring in India, Nepal, Bhutan, Bangladesh, Myanmar, Thailand, China, Laos, Cambodia, Vietnam and Malaysia [5,6]. In India, wild gaurs have been probably domesticated about 2500 years ago [7], mainly for work and meat [8]. Domesticated gaurs are referred to as "gayal" or "mithan" (*Bos frontalis*) and are completely interfertile with their wild relatives [9], which display a karyotype of 2n = 58 [10]. Furthermore, herders breed mithans or cross them with cattle to obtain offspring with enhanced production and performance, however usually only F1 females are fertile and can be used for further breeding purposes.

The global population of wild gaur ranges from 13,000 to 30,000 with a population of mature individuals between 5,200 and 18,000. In the last decades, the number of wild gaurs decreased dramatically due to the loss of suitable habitat (in favour of agriculture and its domestic counterpart), hunting or hybridization with domestic cattle [11]. The latter threat also caused the transmission and outbreak of various devastating diseases, such as foot-and-mouth, rinderpest and anthrax [12]. In Vietnam, the total number of wild gaurs is estimated at a maximum of 500 individuals of which 10% distributed in the Cat Tien National Park, localized close to the Ho Chi Minh City in the south of the country. During 1991–1995, 120 wild gaurs were reported to be killed (more than one generation [1]). Thus, information about the current genetic status of the Vietnamese wild gaur population is important and necessary to develop strategies for conservation and effective long-term management for this species.

Successful amplification and extensive conservation of cattle microsatellite sequences in several species of Bovidae and Cervidae families have been documented in numerous works [13,14], thus allowing possible population genetic studies on related Bovidae species for which microsatellites have not been developed [15-18]. Furthermore, cross-species amplification was also applied to the study of population variations in geographically isolated or endangered species [19,20]. These studies suggest that a characterization of wild gaur, as a member of the subfamily Bovinae, with bovine microsatellite markers is highly pertinent and suitable.

Previous genetic studies were carried out on gaur [21,22], however they were limited to a domesticated group of *Bos frontalis *and only a low number of cattle microsatellites were analyzed. Therefore, the questions about the conservation of cattle microsatellite DNA sequences, as well as the applicability of these markers for population genetic studies in *Bos gaurus *remain open.

The principal aims of this study were (1) to evaluate the applicability and conservation of cattle microsatellite DNA sequences in the wild gaur genome and (2) to estimate the current genetic status of this species in Vietnam.

## Results and discussion

One hundred-thirty cattle microsatellite markers were tested for amplification of genomic DNA from a panel of 11 wild gaurs. Three Brown Swiss cattles (*Bos taurus*) were used as positive control. Although some amplification failures were observed, 90% of the microsatellites from cattle could be successfully amplified by PCR on gaur genomic DNA, of which 68 markers (58.1%) were polymorphic. A total of 264 alleles were detected across the 117 amplified loci with the number of alleles ranging from one to six (Table 1) with a mean of 2.2 alleles per locus. Thirteen microsatellites (10%) distributed on cattle chromosomes 8 (*BM2304*), 10 (*ILSTS005*), 18 (*HAUT14*), 21 (*HEL5*), 24 (*CSSM023*), 26 (*BM1314*, *BMS332 *and *HEL11*), 29 (*BMC2228*), X (*BM6017*, *BMS911 *and *ETH123*) and Y (*INRA189*), respectively, failed to amplify in wild gaur. Notably, the non-amplification of locus *ILSTS005 *indicated the absence of this sequence in both wild gaur and mithan [22]. As expected, all the microsatellite markers could be successfully amplified in the positive control samples (*Bos taurus*), with 92% of them being polymorphic.

**Table 1:** Characterisation of 130 bovine microsatellites tested on a panel of 11 wild gaurs

Marker	Chromosome no. in cattle	Allele size range (bp)	Number of alleles	*H*_*E*_	*H*_*o*_	*PIC*
*AGLA17*	1	217–221	3	0.385	0.273	0.326
*AGLA293*	5	231–231	1	-	-	-
*BL1029*	14	151–155	2	0.091	0.091	0.083
*BL1038*	6	109–109	1	-	-	-
*BL1040*	26	96–108	3	0.255	0.273	0.228
*BL1043*	7	100–104	3	0.177	0.182	0.163
***BL1071***	**13**	**179–195**	**4**	**0.680**	**0.636**	**0.594**
*BL1095*	15	164–174	3	0.385	0.455	0.326
*BL25*	28	171–185	2	0.247	0.273	0.208
BM1314*	26	-	-	-	-	-
*BM1818*	23	264–264	1	-	-	-
*BM1824*	1	187–187	1	-	-	-
*BM1862*	17	201–213	3	0.567	0.727	0.463
*BM188*	26	108–108	1	-	-	-
*BM203*	27	211–213	2	0.312	0.182	0.253
*BM2113*	2	129–129	1	-	-	-
BM2304*	8	-	-	-	-	-
*BM3020*	3	159–159	1	-	-	-
*BM4005*	25	107–107	1	-	-	-
*BM4602*	29	128–130	2	0.519	-	0.373
*BM4621*	6	131–131	1	-	-	-
BM6017*	X	-	-	-	-	-
***BM6425***	**14**	**167–195**	**6**	**0.823**	**0.818**	**0.751**
*BM6438*	1	256–256	1	-	-	-
*BM6465*	3	122–122	1	-	-	-
*BM8139*	1	110–116	3	0.394	0.273	0.344
*BM8151*	18	157–161	3	0.589	0.545	0.476
*BM861*	Y	135–135	1	-	-	-
*BM875*	10	107–119	2	0.519	0.364	0.373
***BMC1410***	**4**	**215–219**	**3**	**0.593**	**0.636**	**0.504**
BMC2228*	29	-	-	-	-	-
*BMC6020*	28	177–177	1	-	-	-
*BMC6021*	X	141–141	1	-	-	-
*BMS1074*	4	157–157	1	-	-	-
***BMS1120***	**20**	**123–137**	**6**	**0.835**	**0.909**	**0.767**
*BMS1128*	20	80–82	2	0.091	0.091	0.083
*BMS1244*	29	103–105	2	0.173	0.182	0.152
*BMS1247*	7	111–121	3	0.537	0.364	0.444
*BMS1282*	20	151–165	4	0.333	0.273	0.302
*BMS1322*	18	117–121	3	0.498	0.091	0.419
*BMS1353*	25	95–103	2	0.368	0.091	0.290
***BMS1355***	**18**	**154–160**	**4**	**0.697**	**0.818**	**0.607**
*BMS1616*	X	65–65	1	-	-	-
*BMS1714*	28	120–122	2	0.416	0.545	0.318
*BMS1825*	17	191–191	1	-	-	-
***BMS1857***	**29**	**155–165**	**4**	**0.675**	**0.545**	**0.575**
*BMS1926*	24	132–136	3	0.394	0.091	0.344
***BMS1928***	**1**	**141–161**	**4**	**0.576**	**0.636**	**0.511**
*BMS1948*	29	93–93	1	-	-	-
*BMS1979*	7	95–99	3	0.498	0.636	0.419
*BMS2213*	18	112–120	2	0.524	0.455	0.375
***BMS2252***	**12**	**158–164**	**4**	**0.697**	**0.455**	**0.604**
*BMS2270*	24	57–63	2	0.485	0.545	0.356
***BMS2526***	**24**	**135–159**	**4**	**0.762**	**0.636**	**0.678**
*BMS2639*	18	160–160	1	-	-	-
*BMS3024*	24	142–142	1	-	-	-
BMS332*	26	-	-	-	-	-
***BMS4015***	**1**	**144–152**	**4**	**0.688**	**0.636**	**0.606**
*BMS424B*	11	256–258	2	0.091	0.091	0.083
*BMS522*	7	134–134	1	-	-	-
*BMS574*	1	131–131	1	-	-	-
*BMS631*	X	146–146	1	-	-	-
*BMS650*	19	141–141	1	-	-	-
*BMS672*	22	143–143	1	-	-	-
*BMS711*	1	102–102	1	-	-	-
*BMS745*	19	109–109	1	-	-	-
*BMS779*	4	191–195	2	0.312	0.364	0.253
BMS911*	X	-	-	-	-	-
*BR4206*	18	110–110	1	-	-	-
*BR4406*	18	114–114	1	-	-	-
*CSRM60*	10	86–114	2	0.368	0.273	0.290
CSSM023*	24	-	-	-	-	-
***CSSM66***	**14**	**182–202**	**3**	**0.593**	**0.727**	**0.504**
*ETH10*	5	207–213	3	0.450	0.455	0.385
***ETH11***	**16**	**204–212**	**4**	**0.688**	**0.636**	**0.593**
*ETH121*	2	182–210	3	0.498	0.455	0.419
ETH123*	X	-	-	-	-	-
*ETH152*	5	198–198	1	-	-	-
*ETH185*	17	219–219	1	-	-	-
***ETH225***	**9**	**145–159**	**3**	**0.636**	**0.636**	**0.524**
***ETH3***	**19**	**127–131**	**3**	**0.654**	**0.545**	**0.553**
HAUT14*	18	-	-	-	-	-
*HAUT24*	22	120–120	1	-	-	-
*HAUT27*	26	145–145	1	-	-	-
***HEL1***	**15**	**108–120**	**3**	**0.628**	**0.636**	**0.519**
HEL11*	26	-	-	-	-	-
*HEL13*	11	193–203	3	0.325	0.364	0.282
HEL5*	21	-	-	-	-	-
***HEL9***	**8**	**146–152**	**4**	**0.610**	**0.545**	**0.533**
*IDVGA59*	26	250–254	3	0.437	0.364	0.360
*IDVGA90*	7	194–194	1	-	-	-
ILSTS005*	10	-	-	-	-	-
*ILSTS006*	7	275–281	2	0.173		0.152
*ILSTS015*	29	265–265	1	-	-	-
*ILSTS017*	X	117–117	1	-	-	-
*ILSTS021*	18	116–116	1	-	-	-
*ILSTS102*	25	146–146	1	-	-	-
***INRA005***	**12**	**135–141**	**4**	**0.697**	**0.818**	**0.600**
***INRA023***	**3**	**207–217**	**4**	**0.710**	**0.636**	**0.623**
***INRA032***	**11**	**169–181**	**5**	**0.753**	**0.727**	**0.674**
*INRA035*	16	108–108	1	-	-	-
***INRA037***	**10**	**126–132**	**4**	**0.727**	**0.636**	**0.637**
***INRA063***	**18**	**173–187**	**5**	**0.758**	**0.636**	**0.675**
*INRA081*	26	145–153	3	0.567	0.545	0.463
*INRA117*	1	91–97	2	0.173	0.182	0.152
***INRA121***	**18**	**114–136**	**4**	**0.710**	**0.545**	**0.615**
*INRA124*	Y	132–132	1	-	-	-
*INRA126*	Y	182–182	1	-	-	-
*INRA133*	6	221–231	3	0.437	0.364	0.360
*INRA183*	27	117–117	1	-	-	-
INRA189*	Y	-	-	-	-	-
*MB054*	18	123–123	1	-	-	-
***MB085***	**15**	**198–202**	**3**	**0.593**	**0.455**	**0.505**
***MHCII***	**23**	**213–225**	**4**	**0.723**	**0.636**	**0.633**
*MM12E6*	9	108–108	1	-	-	-
*RM026*	26	81–81	1	-	-	-
*RM372*	8	128–134	3	0.450	0.364	0.385
*SPS115*	15	253–253	1	-	-	-
***TEXAN10***	**18**	**145–151**	**4**	**0.706**	**0.818**	**0.613**
*TGLA122*	21	166–168	2	0.455	0.455	0.340
*TGLA126*	20	121–125	3	0.498	0.091	0.419
***TGLA179***	**27**	**89–103**	**3**	**0.697**	**0.636**	**0.591**
*TGLA227*	18	72–84	3	0.584	0.455	0.490
*TGLA23*	13	100–104	3	0.567	0.818	0.436
*TGLA49*	1	115–117	2	0.247	0.273	0.208
***TGLA53***	**16**	**151–175**	**5**	**0.701**	**0.727**	**0.606**
***TGLA73***	**9**	**116–126**	**4**	**0.749**	**0.727**	**0.663**
*UWCA25*	13	102–102	1	-	-	-
*XBM11*	X	182–182	1	-	-	-
*XBM7*	X	174–174	1	-	-	-

* = markers not amplified

*H*_*E *_= expected heterozygosity

*H*_*o *_= observed heterozygosity

*PIC *= polymorphism Information Content

The 28 microsatellites with *PIC *value > 0.5 are bold-faced. Information concerning the bovine microsatellite markers used can be acquired from internet sites [32-34].

The applicability of bovine microsatellite markers for genetic studies in several Bovidae species has been reported in different studies and demonstrated extensive genomic conservation of cattle DNA microsatellite sequences during evolution. However, this conservation varies consistently within the Bovidae subfamilies and species (Table 2), as one can also expect by phylogenetic analyses. Additionally, percentage variations of conserved and polymorphic loci also depend on experimental conditions; specifically the number and the identity of the specific set of markers, as well as the number of animals tested play essential roles. This explains the variable levels of marker conservation in water buffalo, goat and sheep obtained from different studies (see Table 2 for references). The average conservation of cattle microsatellite loci across Caprinae species was generally lower than for Bovinae; in fact goat [23] and sheep [13] showed the lowest among all Bovidae. However, these results do not completely account for the experimental differences discussed above, which might influence the finding. With the same set of cattle microsatellites used in this study, our data suggest that *Bos indicus *is more closely related to *Bos taurus *than either *Bos gaurus*, *Poephagus grunniens *or *Pseudoryx nghetinhensis *(Table 2 and references therein). Within the Bovini, a close relationship between wild gaur and banteng (*Bos javanicus*) could be expected, as 90% and 94% of cattle microsatellites were conserved in their genomes, respectively (Table 2). These results were in line with recent taxonomy classifications of Bovidae based on molecular phylogenetic analyses [24,25] and AFLP data [26]. Additionally, genomic conservation of cattle microsatellites has been tested on Cervidae, whereas 73.7% and 74.1% of bovine markers could be successfully amplified in sika deer (*Cervus nippon*) and red deer (*Cervus elaphus*), respectively [14]. Within species of *Bos*, wild gaur showed the lowest proportion of polymorphic markers (Table 2). This finding was in agreement and is possibly related to the small effective population size of Vietnamese wild gaurs, compared to other bovid species. The average allele sizes of most successful amplified markers in wild gaur were smaller compared to those obtained in cattle. This was expected [27] and in agreement with previous studies using cross-species amplification [15,17].

**Table 2:** Genomic conservation of cattle microsatellite loci within the Bovidae and Cervidae families using cross species amplification

Taxon	Species – common name	Conserved loci	Polymorphic loci	References
**Bovidae, Bovinae**				
Bovini, Bovina	*Bos gaurus *– Wild gaur	90%	58.1%	this study
	*Bos indicus *– Zebu	97.6%	87.3%	Nguyen – person. comm.
	*Bos javanicus *– Banteng	94%	75%	Hishida et al. [40]
	*Poephagus grunniens *– Yak	94.6%	94.3%	Nguyen et al. [18]
Bovini, Bubalina	*Bubalus bubalis *– Water buffalo	70%	82%	Moore et al. [19]
		75%	56%	Navani et al. [16]
		85%	57%	Hishida et al. [40]
	*Syncerus caffer *– African buffalo	83%	90%	van Hooft et al. [15]
Bovini, Pseudoryina	*Pseudoryx nghetinhensis *– Saola	96.8%	59.3%	Nguyen et al. [20]
**Bovidae, Caprinae**				
Caprini	*Capra hircus *– Goat	57%	33%	Kemp et al. [23]
		79.4%	81.5%	Kim et al. [17]
	*Ovis aries *– Sheep	58%	67%	de Gortari et al. [13]
		73.4%	42.5%	Slate et al. [14]
Naemorhedini	*Naemorhedus caudatus *– Korean goral	85.3%	55.2%	Kim et al. [17]
**Cervidae, Cervinae**				
Cervus	*Cervus elaphus *– Red deer	74.1%	55.8%	Slate et al. [14]
	*Cervus nippon *– Sika deer	73.7%	37.3%	Slate et al. [14]

The conservation of DNA sequences flanking microsatellites in the sex chromosomes among cattle and wild gaur was evaluated by testing the amplification of nine microsatellite loci, which mapped to BTAX (*BM6017*, *BMC6021*, *BMS1616*, *BMS631*, *BMS911*, *ETH123*, *ILSTS017*, *XBM11 *and *XBM7*) and four additional loci (*INRA124*, *INRA126*, *INRA189 *and *BM861*), which mapped to BTAY. All these sex-specific microsatellite markers were monomorphic. The loci *BM6017*, *BMS911*, *ETH123 *and *INRA189 *failed to amplify sex-chromosome specific DNA in wild gaur. Recently, it has also been reported that locus *BM6017 *could not be amplified in yaks [18]. This could be attributed to the absence of homologous sequences in both species. Moreover, studies demonstrated that *BM861 *and *INRA126 *successfully amplified from both sexes in yak [18,21] and saola (*Pseudoryx nghetinhensis *– [20]), suggesting that they are not Y-specific. These findings indicated that yak and saola X chromosome retained a homologous segment of the Y chromosome, which contains both *BM861 *and *INRA126 *microsatellite markers. Contrary to these studies, we could amplify *INRA124*, *INRA126 *and *BM861 *only in male wild gaurs, indicating that they are Y specific markers in this species. Hanotte et al. [28] also tried to amplify locus *INRA124 *in two males of mithan but failed to obtain an amplification product. Even though we could not find any polymorphism for *INRA124*, *INRA126 *and *BM861*, these three microsatellites were polymorphic in several bovid species, including domestic cattle, bison, mithan, swamp buffalo and yak [21,28]. This may be due to the relative small number (7) of male wild gaurs analyzed, which may have limited the informative content of this marker. In addition, the significant difference in allele size of locus *BM861 *between wild (135 bp) and domestic gaur (mithan, 150–156 bp -[21]) might be explained by the introgressive hybridisation of mithan, leading to the loss of the 135 bp allele from its wild ancestor.

Finally, genotype data collected from the 117 successfully amplified microsatellites were used for genetic studies of the Vietnamese wild gaur population. The expected heterozygosity value per locus across the population varied between 0.091 (*BL1029*, *BMS1128 *and *BMS424B*) and 0.835 (*BMS1120*) (Table 1). Accordingly, markers *BL1029*, *BMS1128 *and *BMS424B *showed the lowest PIC value (0.083), whereas *BMS1120 *had the highest (0.767) with a mean of 0.252. In addition, the observed heterozygosities (*H*_*o*_) ranged from 0.091 to 0.909. Twenty-eight microsatellites (*BL1071*, *BM6425*, *BMC1410*, *BMS1120*, *BMS1355*, *BMS1857*, *BMS1928*, *BMS2252*, *BMS2526*, *BMS4015*, *CSSM66*, *ETH11*, *ETH225*, *ETH3*, *HEL1*, *HEL9*, *INRA005*, *INRA023*, *INRA032*, *INRA037*, *INRA063*, *INRA121*, *MB085*, *MHCII*, *TEXAN10*, *TGLA179*, *TGLA53 *and *TGLA73*; bold-faced in Table 1) showed good level of informativeness, having a PIC value higher than the threshold of 0.5 that is considered the value from which markers begin to be informative and therefore they would be the most suitable for diversity studies. Among these 28 most informative microsatellites, ten (*CSSM66*, *ETH225*, *ETH3*, *HEL1*, *HEL9*, *INRA023*, *INRA032*, *INRA037*, *INRA063 *and *TGLA53*) are also in the FAO standard panel of 30 microsatellites for diversity studies, allowing the study of introgression.

The average observed heterozygosity value (*H*_*o *_= 0.269) was lower than the average expected heterozygosity (Nei's unbiased mean heterozygosity; *H*_*E *_= 0.298) and this difference was statistically significant. Eleven (*BM4602*, *BMS1322*, *BMS1353*, *BMS1926*, *ILSTS006*, *INRA037*, *INRA063*, *MHCII*, *TEXAN10*, *TGLA126 *and *TGLA73*) out of 117 loci (9.4%) showed significant deviation from the Hardy-Weinberg equilibrium at *p *< 0.05. Over all loci, departure from Hardy Weinberg equilibrium was statistically highly significant (*p *< 0.001), reflecting the deviation in the direction of heterozygote deficit. These results indicate a frequent portion of homozygous individuals in the Vietnamese wild gaur population, resulting in an inbreeding coefficient value [F = (*H*_*E *_- *H*_*o*_)/*H*_*E*_] of 0.10. Deviations from Hardy-Weinberg equilibrium of the population studied might be the results of inbreeding, but could also have been caused by the presence of non-amplifying (null) alleles, which could have contributed to the heterozygote deficiencies. In addition, the low average heterozygosity of wild gaurs may also be the consequence of the use of cattle derived microsatellite markers, which are expected to perform less in related species, having a higher fraction of null alleles and being less polymorphic.

## Conclusion

The degree of polymorphism in the high number of microsatellite markers tested provides important information about the current genetic status of Vietnamese wild gaur. Its small population size would be dramatically adversely affected by high inbreeding and genetic drift. Therefore, the use of cattle microsatellites is adequate and recommended for further population genetic analyses, aimed to develop effective long-term conservation plans and strategies for this threatened species in Asia, especially in Vietnam. The reported low level of genetic diversity in wild gaur possibly reflects a bottleneck effect following the dramatic population reduction that occurred in this country during 1991–1995.

## Methods

### Sample collection

Eleven wild gaur samples (7 males and 4 females) were randomly collected in South Vietnam from the Chu Mom Ray Nature Reserve, Kon Tum province and Thao Cam Vien (Zoo and Botanical Garden), Ho Chi Minh City. Genomic DNA was extracted from tissue samples, fibroblast cells and bone fragments following standard methods [29,30] with minor modifications. DNA from three Brown Swiss cattles (*Bos taurus*) was obtained from EDTA-anticoagulated whole blood [31] and used as positive control.

### Microsatellite analysis

The same set of 130 bovine microsatellite markers analyzed by Nguyen et al. [18], excluded *BPLP*, and distributed across the entire cattle genome (Table 1) was tested for PCR amplification on wild gaur genomic DNA. The primer pairs, which show extensive polymorphism in cattle, were selected from internet sites [32-34]. The forward primer of each microsatellite was 5'-labeled with either *FAM*, *JOE*, *TAMRA*, *HEX *or *TET *fluorescent tag. PCR amplification was carried out, as described by Nguyen et al. [18], in a total reaction volume of 25 μl containing 20–30 ng DNA template, 1× PCR buffer (10 mM Tris-HCl, pH 8.3, 50 mM KCl, 1.5 mM MgCl2), 1.25 mM of dNTP mix, 20 μM of each primer and 1.25 units of *Taq *polymerase (SIGMA, Buchs, Switzerland). Samples were cycled in a PCR Express Machine (Thermocycler PCR Express, Hybaid) at 95°C for 5 min, followed by 35 cycles of 95°C for 30 s, 52–60°C annealing temperature (depending on the microsatellite used) for 30 s and 72°C for 30 s. The final elongation was at 72°C for 7 min. Gel electrophoresis was performed with a 377 ABI sequencer (Applied Biosystems, Rotkreuz, Switzerland) with Genescan-350 TAMRA or ROX as internal standards. Fragment sizing and analysis were done using ABI 672 Genescan software and Genotyper (version 2.1) software (Applied Biosystems).

### Statistical analysis

Genotypes were assigned for each individual based on allele size data. Allele frequencies, expected heterozygosity (*H*_*E *_= 1 - ∑ P_i_^2^, where P_i _= frequency of allele i), observed heterozygosity (*H*_*o*_) for all loci were computed using the Microsatellite Toolkit version 3.1 [35]. Genetic diversity was estimated according to Nei [36], using the average heterozygosity across all loci. Probability tests of Hardy-Weinberg equilibrium [37] based on Markov chain approaches (5000 iterations) were performed using the GENEPOP package version 3.4 [38]. The polymorphism information content (PIC) was calculated using the following formula:

PIC=1−∑i=1Pi2−∑i=1∑j=i+1Pi2Pj2
 MathType@MTEF@5@5@+=feaafiart1ev1aaatCvAUfKttLearuWrP9MDH5MBPbIqV92AaeXatLxBI9gBaebbnrfifHhDYfgasaacPC6xNi=xI8qiVKYPFjYdHaVhbbf9v8qqaqFr0xc9vqFj0dXdbba91qpepeI8k8fiI+fsY=rqGqVepae9pg0db9vqaiVgFr0xfr=xfr=xc9adbaqaaeGacaGaaiaabeqaaeqabiWaaaGcbaGaeeiuaaLaeeysaKKaee4qamKaeyypa0JaeGymaeJaeyOeI0YaaabuaeaacqqGqbaudaqhaaWcbaGaeeyAaKgabaGaeGOmaidaaaqaaiabbMgaPjabg2da9iabigdaXaqab0GaeyyeIuoakiabgkHiTmaaqafabaWaaabuaeaacqqGqbaudaqhaaWcbaGaeeyAaKgabaGaeGOmaidaaOGaeeiuaa1aa0baaSqaaiabbQgaQbqaaiabikdaYaaaaeaacqqGQbGAcqGH9aqpcqqGPbqAcqGHRaWkcqaIXaqmaeqaniabggHiLdaaleaacqqGPbqAcqGH9aqpcqaIXaqmaeqaniabggHiLdaaaa@50AE@

where P_i _and P_j _are frequencies of i^th ^and j^th ^alleles [39].

## Authors' contributions

TTN and SG prepared the DNA samples, performed the microsatellite analysis and drafted the manuscript. LCB carried out the statistical analysis and drafted the manuscript. PV, GS and JPR coordinated the analyses and helped in drafting the manuscript. JCM and BXN conceived and supervised the entire study. All authors read and approved the final manuscript.
